# Celestial Insights: Unraveling the Role of miR-3682-3p in Hepatocellular Carcinoma

**DOI:** 10.14309/ctg.0000000000000690

**Published:** 2024-04-25

**Authors:** Pei-Dong Miao, Ying Li, Yu-Dong Jiang

**Affiliations:** 1 Dalian No.3 People's Hospital, Department of Interventional Radiology, Dalian, People's Republic of China;; 2 Dalian 7th People's Hospital, Dalian, Liaoning Province, China.

**Keywords:** miR-3682-3p, miRNA, hepatocellular carcinoma, biomarkers, tumor microenvironment, prognosis, cancer therapeutics, bioinformatics analysis

## Abstract

Hepatocellular carcinoma (HCC) remains a formidable oncological challenge, calling for innovative therapeutic strategies to improve patient outcomes. MicroRNAs have emerged as key regulators in cancer, and miR-3682-3p shows potential as a diagnostic and prognostic biomarker in HCC. We conducted a comprehensive study to uncover its role in HCC biology, revealing dysregulation and clinical associations. Target gene analysis provided insights into potential molecular mechanisms. Moreover, we explored its impact on the tumor microenvironment, immune cell infiltration, and therapy responses. Our findings highlight miR-3682-3p as a promising candidate for further investigations and potential therapeutic strategies in HCC management.

## INTRODUCTION

Hepatocellular carcinoma (HCC), a prevalent and life-threatening liver cancer, continues to challenge global healthcare systems (1). Understanding the molecular intricacies driving HCC's development and progression is crucial for improving patient outcomes. Recently, microRNAs (miRNAs) have emerged as key players in cancer biology, influencing tumor growth, spread, and response to treatment (2).

MiRNAs are tiny RNA molecules that regulate gene expression post-transcriptionally (3). Their disrupted function has been linked to altered expression of critical genes involved in cancer-related processes (2). Among these miRNAs, miR-3682-3p has gained attention as a potential diagnostic and prognostic marker in HCC (4,5). Although previous studies have touched on its significance, a comprehensive exploration of its role in liver cancer biology, encompassing expression patterns, regulatory targets, and interactions within the tumor microenvironment is still needed.

This study takes a holistic approach to investigate miR-3682-3p in liver cancer. Leveraging bioinformatics analyses, survival assessments, and functional investigations, our aim was to uncover its diagnostic utility, prognostic value, and underlying mechanisms. We also explored its relationships with immune cell presence, therapy responses, and its influence on critical signaling pathways in HCC.

## MATERIALS AND METHODS

### Data acquisition and target miRNA selection

The data used in this study were procured from The Cancer Genome Atlas database (TCGA, downloaded from the Genomic Data Commons Data Portal at https://portal.gdc.cancer.gov/, https://portal.gdc.cancer.gov/). Specifically, miRNA data for 33 distinct tumor types included in the TCGA database were downloaded, along with mRNA and clinical data from the liver hepatocellular carcinoma (LIHC) data set. Initially, differentially expressed miRNAs between tumor patients and normal samples within the LIHC data set were calculated. Subsequently, univariate survival analysis and independent prognosis factor analysis were used to further filter the miRNAs. Ultimately, miRNAs with an area under the curve (AUC) >0.6 for the prediction of liver cancer patients' prognosis were identified.

### Correlation between miR-3682-3p and clinical phenotypes of liver cancer

For subsequent analysis, miR-3682-3p was selected. Its expression differences between patients and normal samples in 33 different tumor types, with a specific focus on liver cancer, were evaluated. The relationship with the survival of patients with liver cancer was assessed using Kaplan-Meier curves and univariate cox proportional hazards model (COX) regression analysis. The potential of miR-3682-3p as an independent prognostic factor for patients with liver cancer was confirmed through univariate and multivariate regression. Diagnostic receiver operating characteristic (ROC) curves and prognostic ROC curves were also used to assess the diagnostic and prognostic capabilities of miR-3682-3p, respectively. The associations of miR-3682-3p with other prevalent clinical phenotypes of liver cancer were presented through correlation analysis.

### MiR-3682-3p–related mRNAs and functions

The mRNAs regulated by miR-3682-3p were predicted using the TargetScan website (https://www.targetscan.org/vert_80/) (6). Subsequently, the mRNAs under the regulatory control of miR-3682-3p were identified by evaluating the relationship between their expression levels and miR-3682-3p expression levels, with a criterion of *P* value <0.05 and a negative correlation. Furthermore, the biological functions executed by these identified mRNAs were forecasted through the employment of the gene set enrichment analysis (GSEA) method (7).

### Correlation between miR-3682-3p and liver cancer immunity

Through the utilization of software tools such as estimate (8), CIBERSORT (9), and tumor immune dysfunction and exclusion (10), the simulation of the tumor microenvironment and immune cell infiltration in patients with liver cancer was efficiently achieved using mRNA data from the LIHC data set. This facilitated the prediction of the response of patients with liver cancer to immunotherapy. Subsequently, the correlations between miR-3682-3p and these factors were computed.

The current study was approved by the Dalian NO. 3 People's Hospital. This study utilized publicly available bioinformatics data, including genome sequences and expression data from the NCBI database and other public resources. We confirm compliance with relevant usage licenses for all data utilized, and adhere strictly to applicable regulations and ethical standards throughout data analysis and result reporting. As this study did not involve experiments on humans or animals, ethical review was not required. We respect the privacy policies of all data sources and ensure the implementation of appropriate security measures during data processing and storage to safeguard data privacy and security.

## RESULTS

### Identification of miR-3682-3p

Within the confines of the LIHC data set, a comprehensive count of 207 miRNAs manifested discernible expression disparities between the patient and control cohorts, adhering to the established criterion (threshold: *P* value<0.05, |logFC|>1). Noteworthy among these, a constellation of 7 miRNAs (namely, miR-7-5p, miR-139-5p, miR-9-5p, miR-4661-5p, miR-551a, miR-3682-3p, and miR-3677-3p) exhibited statistically significant associations with the prognostic trajectories of patients with liver cancer. These associations were delineated through the application of both Kaplan-Meier survival analysis and univariate COX regression, maintaining the prescribed threshold for statistical significance (criterion level: *P* value <0.05) in both analytical modalities. Importantly, within this subset of miRNAs, a notable quintet (miR-7-5p, miR-9-5p, miR-4661-5p, miR-3682-3p, and miR-3677-3p) emerged as autonomous prognostic determinants for patients with liver cancer, evincing their influence on patient outcomes independent of confounding factors. In summation, a total of 4 miRNAs (specifically, miR-9-5p, miR-4661-5p, miR-3682-3p, and miR-3677-3p) stood out as possessing the inherent capability to prognosticate the trajectory of liver cancer, a notion supported by their impressive AUC values surpassing the threshold of 0.6 (see Supplementary Digital Content 1, TableS1-4, http://links.lww.com/CTG/B90).

### Relationship between miR-3682-3p and clinical phenotypes of liver cancer

**Figure 1.**
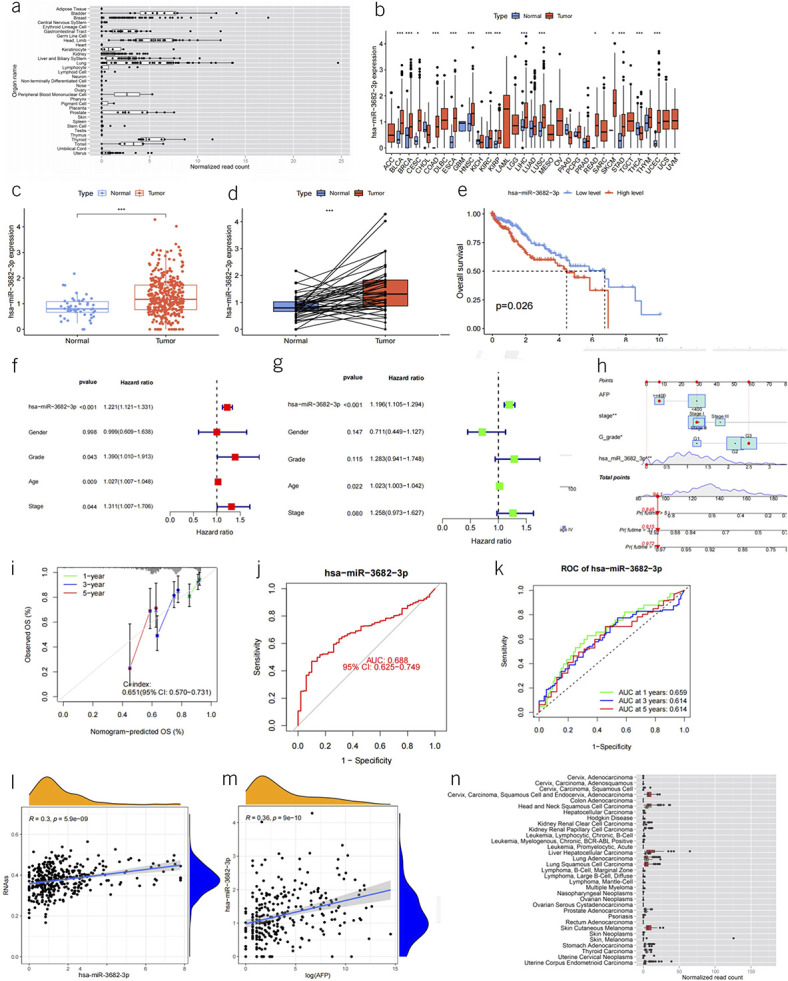
(**a**) Box plot depicting the expression levels of miR-3682-3p across various organs (picture from miRGator). (**b**) Box plot illustrating the expression levels of miR-3682-3p in 33 data sets from the TCGA database (red represents disease group, and blue represents control group). (**c**) Box plot showcasing the expression levels of miR-3682-3p in tumor and control groups within the LIHC data set. (**d**) Box plot comparing the expression levels of miR-3682-3p in tumor patients and paired normal control samples within the LIHC data set. (**e**) Kaplan-Meier curves displaying the survival distribution of miR-3682-3p high and low expression groups within the LIHC data set. (**f**, **g**) Forest plots illustrating the results of univariate and multivariate Cox regression analyses for independent prognostic factors, including miR-3682-3p, gender, G grade, AFP, and stage. (**h**) Nomogram representing a prognostic model built using miR-3682-3p, G grade, AFP, and stage to predict 1-, 3-, and 5-year survival rates of patients with liver cancer. (**i**) Calibration curves for the nomogram's predictions of 1-, 3-, and 5-year survival rates. (**j**) ROC curve depicting the ability of miR-3682-3p expression levels to predict liver cancer diagnosis. (**k**) ROC curve illustrating the capability of miR-3682-3p expression levels to predict liver cancer prognosis. (**l**) Scatter plot with trendline demonstrating the relationship between miR-3682-3p expression and RNA stability score (RNAs) in patients with liver cancer. (**m**) Scatter plot with trendline showcasing the relationship between miR-3682-3p expression and AFP levels in patients with liver cancer. (**n**) Box plots of miR-3682-3p expression levels in tumor patients (red) compared with normal samples (green) (a part of picture from miRGator). AFP, alpha-fetoprotein; LIHC, liver hepatocellular carcinoma; ROC, receiver operating characteristic; TCGA, The Cancer Genome Atlas database.

MiR-3682-3p exhibits elevated expression within the liver (miRGator v3.0, http://mirgator.kobic.re.kr/) (Figure 1a). Among the 33 tumor types included in the TCGA data set, except for 10 types where miRNA data sets lack control samples (adrenocortical carcinoma, diffuse large B-cell lymphoma, acute myeloid leukemia, low-grade glioma, mesothelioma, ovarian serous cystadenocarcinoma, sarcoma, testicular germ cell tumors, uterine carcinosarcoma, and uveal melanoma), miR-3682-3p demonstrates differential expression in data sets from 15 tumor types (15/23) (Figure 1b). Most instances show upregulation in tumor samples (except for THCA, where it is upregulated in control samples). Within the LIHC data set, irrespective of paired *t* tests, the expression levels in the tumor group are significantly higher than those in the control group (*P* < 0.05) (Figure 1c,d,n). It exhibits a significant association with the survival of patients with liver cancer (*P* = 0.026) (Figure 1e). Notably, it emerges as an independent prognostic factor for patients with liver cancer in both univariate and multivariate COX regression analyses (Figure 1f,g).

In terms of diagnostic predictions for liver cancer, the AUC reaches 0.688 (Figure 1j). When used for predicting the 1-, 3-, and 5-year survival rates of patients with liver cancer, the AUC values are 0.659, 0.614, and 0.614, respectively (Figure 1k). Its expression levels correlate with alpha-fetoprotein (AFP) levels in patients with liver cancer (correlation coefficient 0.36, *P* value < 0.001) (Figure 1m). However, miR-3682-3p expression is unrelated to clinical phenotypes of patients with liver cancer, including gender, person neoplasm cancer status, family cancer history, G grade, vascular tumor cell type, Child-Pugh, pathologic stage, T stage, N stage, and M stage (*P* ≥ 0.05) (Table 1). In addition, the expression level of miR-3682-3p is positively correlated with liver cancer stemness (correlation coefficient *R* = 0.3, *P* = 5.9e−09) (Figure 1l). We established a prognostic model using a combination of AFP levels, miR-3682-3p expression, stage, and G grade. This model was then used to generate a nomogram for prognostic assessment (Figure 1h). Preliminary validation of this model revealed a concordance index of 0.651, indicating a reasonable degree of predictive accuracy. In addition, the calibration curves indicated a satisfactory alignment between predicted outcomes and actual observations (Figure 1i).

**Table 1.** Relationships between miR-3682-3p expression and clinical phenotypes of patients with liver cancer

Covariates	Group	Total	Low	High	χ^2^	*P* value
Gender	Female	119 (31.99%)	53 (28.49%)	66 (35.48%)	1.7793	0.1822
	Male	253 (68.01%)	133 (71.51%)	120 (64.52%)		
Cancer status	Tumor-free	233 (67.54%)	113 (65.7%)	120 (69.36%)	0.3748	0.5404
	With tumor	112 (32.46%)	59 (34.3%)	53 (30.64%)		
Relative family cancer history	No	211 (65.73%)	103 (62.8%)	108 (68.79%)	1.0237	0.3117
	Yes	110 (34.27%)	61 (37.2%)	49 (31.21%)		
G grade	G1	55 (14.95%)	27 (14.75%)	28 (15.14%)	6.6756	0.083
	G2	176 (47.83%)	99 (54.1%)	77 (41.62%)		
	G3	124 (33.7%)	52 (28.42%)	72 (38.92%)		
	G4	13 (3.53%)	5 (2.73%)	8 (4.32%)		
Vascular tumor cell type	Macro	17 (5.36%)	8 (4.94%)	9 (5.81%)	0.7739	0.6791
	Micro	94 (29.65%)	45 (27.78%)	49 (31.61%)		
	None	206 (64.98%)	109 (67.28%)	97 (62.58%)		
Child-Pugh	A	220 (90.91%)	118 (92.19%)	102 (89.47%)	1.7883	0.409
	B	21 (8.68%)	9 (7.03%)	12 (10.53%)		
	C	1 (0.41%)	1 (0.78%)	0 (0%)		
Pathologic stage	I	172 (49.43%)	90 (51.43%)	82 (47.4%)	0.7584	0.8594
	II	86 (24.71%)	41 (23.43%)	45 (26.01%)		
	III	85 (24.43%)	42 (24%)	43 (24.86%)		
	IV	5 (1.44%)	2 (1.14%)	3 (1.73%)		
T	T1	182 (49.32%)	96 (52.46%)	86 (46.24%)	2.8813	0.4103
	T2	94 (25.47%)	44 (24.04%)	50 (26.88%)		
	T3	80 (21.68%)	39 (21.31%)	41 (22.04%)		
	T4	13 (3.52%)	4 (2.19%)	9 (4.84%)		
N	N0	254 (98.45%)	122 (98.39%)	132 (98.51%)	0	1
	N1	4 (1.55%)	2 (1.61%)	2 (1.49%)		
M	M0	269 (98.53%)	136 (99.27%)	133 (97.79%)	0.2612	0.6093
	M1	4 (1.47%)	1 (0.73%)	3 (2.21%)		

**Figure 2.**
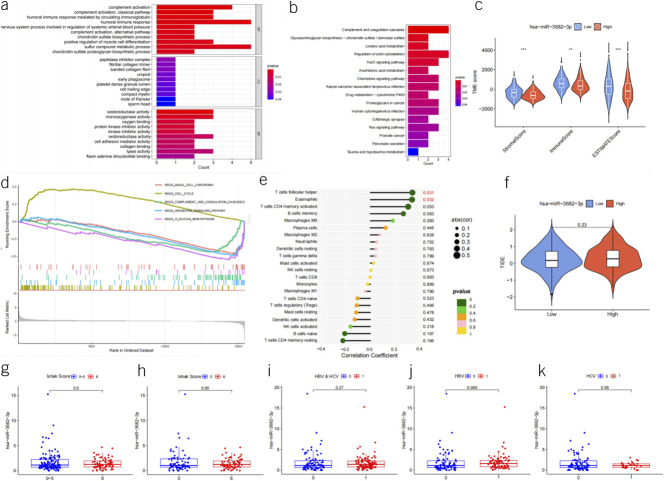
(**a**) Bar plot showing the GO enrichment analysis of the 52 potential target genes that may be regulated by miR-3682-3p. (**b**) Bar plot illustrating the KEGG pathway enrichment analysis of the 52 potential target genes that may be regulated by miR-3682-3p. (**c**) Violin plot depicting the tumor microenvironment (TME) score distribution between the high and low expression groups based on the median miR-3682-3p expression level. (**d**) Gene set enrichment analysis (GSEA) plot displaying the differential gene expression analysis between the high and low miR-3682-3p expression groups. (**e**) Lollipop plot representing the relationship between miR-3682-3p expression levels and the predicted immune cell composition in patients with liver cancer using CIBERSORT. (**f**) Violin plot showing the tumor immune dysfunction and exclusion (TIDE) score distribution between the high and low miR-3682-3p expression groups. (**g**) Box plot depicting the expression levels of miR-3682-3p in patients with Ishak scores ranging from 0 to 5 compared with those with an Ishak score of 6. (**h**) Box plot illustrating the expression levels of miR-3682-3p in patients with Ishak score 0 compared with those with Ishak score 6. (**i**) Box plot representing the expression levels of miR-3682-3p in liver cancer patients with hepatitis B or C virus infections compared with those with non–viral hepatitis-related liver cancer. (**j**) Box plot showing the expression levels of miR-3682-3p in patients with liver cancer with hepatitis B compared with those with non–viral hepatitis-related liver cancer. (**k**) Box plot displaying the expression levels of miR-3682-3p in patients with liver cancer with hepatitis C compared with those with non–viral hepatitis-related liver cancer. GO, gene ontology; KEGG, Kyoto encyclopedia of genes and genomes.

To compare the expression levels of miR-3682-3p in patients with liver cancer with or without cirrhosis, we stratified all patients in the LIHC data set based on the Ishak score. We compared the Ishak 0 score group (76 samples) vs the Ishak 6 score group (72 samples) and the Ishak 0–5 score group (146 samples) vs the Ishak 6 score group (72 samples). The results showed that there were no significant differences in the expression levels of miR-3682-3p between these groups (*P* > 0.05) (Figure 2g,h).

By comparing the expression levels of miR-3682-3p in patients with liver cancer with or without viral hepatitis (including hepatitis B virus and hepatitis C virus) in the LIHC data set, we found no significant differences between these groups (*P* > 0.05). We even stratified patients based on hepatitis B virus (76 patients), hepatitis C virus (28 patients), and both hepatitis B and C virus infections (104 patients) for 3 separate comparisons against non–viral hepatitis-related patients with liver cancer (92 samples), and the results showed no statistical significance (Figure 2i,j,k).

### Functional mechanism of miR-3682-3p

The TargetScan database predicted a total of 1,388 target genes potentially regulated by miR-3682-3p (see Supplementary Digital Content 2, http://links.lww.com/CTG/B91). Within the LIHC data set, a comprehensive correlation analysis was conducted to identify 52 genes for which the expression may be modulated by miR-3682-3p (*P* < 0.05, Pearson correlation coefficient <0). These genes encompass CFHR4, CYYR1, PLA2G5, TMEM154, CXCL12, HOPX, TPRG1, CYP2E1, TACR1, TIGD2, GABARAPL1, ITPRIP, TIAM1, NFASC, EXPH5, SLIT3, ENDOD1, MMAA, GALNT15, XYLT1, ANKRD29, NOVA2, C1RL, ITGA11, SLC14A1, TCF21, RBMS3, MICAL2, PLAT, CDKN1A, TOX, BST1, OCIAD2, CDC37L1, SOD2, CHST7, CLYBL, ECHDC2, GNG11, BNC2, SERTAD1, C3, FMO3, C7, WLS, GLYAT, TTPA, CLEC14A, BMP10, PLLP, and LUM (see Supplementary Digital Content 1, Table S5, http://links.lww.com/CTG/B90). The primary functions of these genes are outlined in Figure 2a,b.

By stratifying patients with liver cancer into high and low expression groups based on the median miR-3682-3p expression level, a calculation of differentially expressed mRNAs between these groups was performed. Subsequently, functional analysis using GSEA was executed to unveil the biological implications of these mRNA alterations. The outcome of this analysis is summarized in Figure 2d and see Supplementary Digital Content 1, Table S6, http://links.lww.com/CTG/B90.

### MiR-3682-3p and tumor microenvironment and treatment

Computation based on the ESTIMATE prediction results suggests that miR-3682-3p may be associated with liver cancer patients' StromalScore, ImmuneScore, and ESTIMATEScore (Figure 2c). Calculations using the CIBERSORT prediction results indicate that miR-3682-3p might only be correlated with the expression levels of 2 cell types: T cells follicular helper and eosinophils (both are positively correlated) (Figure 2e). Evaluations of the tumor immune dysfunction and exclusion prediction results indicate that miR-3682-3p's presence may not significantly relate to the benefits of immune therapy for patients with liver cancer (*P* = 0.23) (Figure 2f). Furthermore, computations based on the oncoPredict software results suggest that the expression level of miR-3682-3p may be associated with the sensitivity to 91 drugs (*P* < 0.05) (see Supplementary Digital Content 1, Table S7, http://links.lww.com/CTG/B90).

## DISCUSSION

### The significant role of miRNA in tumors

MiRNAs play a crucial role in influencing the 6 essential characteristics of malignant cells: self-sufficiency in growth signals, insensitivity to antigrowth signals, evasion from apoptosis, limitless replicative potential, angiogenesis, and invasion and metastases (2). Initially, it was believed that miRNA expression was generally downregulated in tumor tissues (11), aligning with the observation of the loss of differentiation capacity in tumor cells. However, later studies gradually revealed that the changes in miRNA within tumors are not fixed (12). In our research, among the 207 differentially expressed miRNAs calculated using the limma package (*P* < 0.05), 83 miRNAs were downregulated (logFC <0), whereas the majority (124 miRNAs) were upregulated (logFC >0) in tumor tissues. The mechanisms of miRNA in tumors are intricate, sometimes acting as tumor suppressors and other times promoting tumorigenesis (13). Remarkably, miRNAs can enter circulation through extracellular vesicles, enabling them to reach target organs systemically for regulation (14). This characteristic gives miRNAs significant diagnostic value, particularly in early tumor detection (15). In our study, the use of miR-3682-3p alone for diagnosing liver cancer yielded an AUC of 0.688. Moreover, when predicting the 1-, 3-, and 5-year survival rates of patients with liver cancer based solely on miR-3682-3p levels, the AUC remained above 60%. As miRNAs often regulate multiple mRNAs simultaneously, manipulating mRNA through miRNA regulation is often more effective (16), providing miRNAs with promising applications in the field of tumor treatment (17). Our study also discovered that miR-3682-3p could influence the sensitivity of as many as 91 antitumor drugs.

### Current research achievements on miR-3682-3p

In recent years, several studies on the relationship between miR-3682-3p and liver cancer have been published. These studies primarily focus on which pathways miR-3682-3p regulates in liver cancer, including the RAS-MEK1/2-ERK1/2 pathway (18), FOXO3/PI3K/AKT/c-Myc pathway (19), miR-3682-3p-PHLDA1-FAS pathway (20), and PTEN/PI3K/AKT/beta-catenin pathway (21). Some researchers have investigated the correlation between miR-3682-3p expression levels in liver cancer tissues and patient survival, concluding that high expression of miR-3682-3p is a poor prognostic factor in HCC patients (5). However, comprehensive studies using bioinformatics methods to explore the expression patterns of miR-3682-3p in pan-cancer, its relationships with various clinical phenotypes in liver cancer, the potential biological mechanisms it may exert in patients with liver cancer, and its relevance to immune cell infiltration and immune therapy in patients with liver cancer remain unreported. This study fills this knowledge gap by providing a comprehensive investigation in these aspects.

### The relationship between miR-3682-3p and AFP

AFP, a biomarker produced initially by the yolk sac during the early stages of pregnancy, is secreted by the fetal liver and gastrointestinal tract starting from the fourth week of pregnancy, persisting throughout embryonic development (22). It is one of the earliest and widely used serum markers in clinical practice (23) and plays a significant role in liver cancer diagnosis (24), classification (25), treatment efficacy evaluation (26), and prognosis (27). Our research revealed a positive correlation between miR-3682-3p and AFP expression levels. Hence, we infer that miR-3682-3p does not directly regulate the AFP gene. This hypothesis aligns with the predictions from the TargetScan database (https://www.targetscan.org/vert_80/) (28). A query in the Kyoto encyclopedia of genes and genomes (KEGG) database indicates that the AFP gene is primarily involved in the Hippo signaling pathway (hsa04390, website: https://www.kegg.jp/entry/pathway+hsa04390) (29). This pathway encompasses 157 genes, and there is no intersection between the 51 genes directly regulated by miR-3682-3p and the genes in the Hippo pathway. The GENE database under NCBI, which collects genes directly related to AFP (https://www.ncbi.nlm.nih.gov/gene/174), includes a total of 24 genes, and there is no overlap with the 51 genes we identified as being directly regulated by miR-3682-3p. Hence, we believe that miR-3682-3p may influence the expression of the AFP gene through a more complex mechanism, warranting further in-depth exploration.

### The biological functions of miR-3682-3p in liver cancer

According to the TargetScan website, the 51 target genes predicted to be directly regulated by miR-3682-3p show a significant negative correlation with the expression level of miRNA (*P* < 0.05). KEGG enrichment analysis of these genes suggests that miR-3682-3p may play a role in various biological functions in liver cancer (see Supplementary Digital Content 1, Table S8, http://links.lww.com/CTG/B90), including:

1. Complement and coagulation cascades: In HCC, aberrant activation of the coagulation and complement systems may lead to inflammation, thrombus formation, and alterations in the tumor microenvironment, thereby influencing tumor growth, invasion, and metastasis. There exists a close relationship between tumors and coagulation dysfunction (30). Thrombosis, especially portal vein thrombosis, is a common complication in HCC, often difficult to manage, and significantly impacts patient survival (31,32). Certain coagulation-related biomolecules, such as PIVKA-II, have become commonly used tumor markers in clinical practice for AFP-negative patients (33). Therefore, understanding the mechanisms underlying coagulation dysfunction in HCC and identifying risk factors for predicting thrombosis in HCC patients have crucial clinical significance. The next step, if we can validate the relationship between miR-3682-3p and thrombosis in HCC patients through biological experiments, could have a substantial impact on HCC treatment and prognosis.
2. Glycosaminoglycan biosynthesis: chondroitin sulfate/dermatan sulfate: Abnormal synthesis of glycosaminoglycans may be related to cell adhesion, signal transduction, and metastasis in liver cancer, influencing the interaction between tumor cells and the extracellular matrix (34). Interestingly, an important function of glycosaminoglycans is their ability to bind to enzymes or enzyme inhibitors, thereby influencing coagulation function (35).
3. Linoleic acid metabolism: The linoleic acid metabolism pathway may play a role in regulating inflammation, cell proliferation, and growth factor signaling, all of which are closely associated with liver cancer development (36,37).
4. Regulation of actin cytoskeleton: The reconstruction of the cell cytoskeleton is crucial for the invasion and migration of liver cancer cells (38). This pathway may be involved in the regulation of tumor cell morphology and motility (39).
5. FoxO signaling pathway: FoxO proteins participate in regulating the cell cycle, apoptosis, and antioxidant responses (40,41). In liver cancer, the FoxO pathway may play a role in regulating tumor cell survival, proliferation, and metabolism (42).
6. Arachidonic acid metabolism: The arachidonic acid metabolism pathway is related to inflammation, tumor growth, and invasion (43). Abnormal metabolism in this pathway may enhance the proliferation and metastatic potential of liver cancer cells (43).
7. Chemokine signaling pathway: Chemokines play a crucial role in the tumor microenvironment, influencing the migration, invasion, and immune escape of liver cancer cells (44).

### MiR-3682-3p and tumor microenvironment, as well as immune cell infiltration in liver cancer

Cancer cells do not act alone; they interact extensively with the extracellular matrix and stromal cells, forming the major components of the tumor microenvironment (45). Liver cancer often arises in the context of liver inflammation or cirrhosis, characterized by significant fibrous tissue proliferation (1,46). Thus, the relationship between liver cancer development and the tumor microenvironment is highly significant (47). In this study, we found that miR-3682-3p is significantly associated with StromalScore, ImmuneScore, and ESTIMATEScore in liver cancer. This suggests that miR-3682-3p may play a potential role in the early stages of liver cancer development, including during the period of liver cirrhosis. However, contrasting these findings, when applying the CIBERSORT software to predict immune cell infiltration in LIHC data, a surprising observation emerged: Only a small subset of samples demonstrated statistically significant results (42/425, *P* < 0.05). The limited sample size undoubtedly places constraints on the credibility of our subsequent investigations. We discovered that only T cells follicular helper and eosinophils cell content correlated with miR-3682-3p expression. T cells follicular helper, a specific subset of CD4^+^ T cells, play a crucial role in promoting immune responses by facilitating B-cell reactions within the body (48,49). In tumors, the function of T cells follicular helper may be essential for immune surveillance and tumor antigen-specific immune responses (50). Studies suggest that the quantity and function of T cells follicular helper in patients with liver cancer may be impaired, potentially leading to restricted immune responses and interfering with the ability to prevent tumor development through humoral immunity (51). The primary function of eosinophils includes participation in allergic reactions, clearance of parasitic infections, and modulation of inflammatory responses; however, their role in tumors remains a subject of debate (52,53). One possibility is that eosinophils are recruited to the liver by follicular helper T cells through CXCL12 (54). In conclusion, more effective methods are needed to predict immune cell infiltration in liver cancer tissue, leading to a better understanding of the relationship between miR-3682-3p and immune cell infiltration in liver cancer.

### MiR-3682-3p: unrelated to cirrhosis or hepatitis

Liver cancer is closely related to hepatitis and cirrhosis (55). Therefore, is the elevated expression of miR-3682-3p in patients with liver cancer primarily based on its association with cirrhosis or viral hepatitis? Through the analysis of TCGA database LIHC data, this study provides a negative answer: The expression level of miR-3682-3p is not correlated with cirrhosis or viral hepatitis. Thus, we conclude that the expression level of miR-3682-3p is unrelated to cirrhosis and hepatitis but is associated with liver cancer.

In recent years, with the emergence of a large number of tyrosine kinase inhibitors (TKIs) and immune checkpoint inhibitors, there are more treatment options for liver cancer. Studies have confirmed that a combination therapy strategy involving TKIs and immune checkpoint inhibitors has significantly higher antitumor efficacy (56). Alongside this, there is growing consideration of resistance to these new antitumor drugs. Some studies suggest that noncoding RNAs, including circRNAs and miRNAs, play crucial roles in the resistance process to TKIs and other antitumor drugs (57,58). The specific role of miR-3682-3p in liver cancer resistance warrants further investigation.

### MiR-3682-3p in HCC drug treatment

Among the 91 drugs predicted by the oncoPredict software to potentially correlate with miR-3682-3p expression, the sensitivity of the majority of these drugs is positively correlated with miR-3682-3p expression (87/91) (see Supplementary Digital Content 1, Table S7, http://links.lww.com/CTG/B90). This association may be linked to the enrichment of genes regulated by miR-3682-3p in the cytochrome P450 metabolic pathway (hsa00982, see Supplementary Digital Content 1, Table S8, http://links.lww.com/CTG/B90). Notably, clinically used HCC drugs such as gemcitabine, epirubicin, vincristine, oxaliplatin, sorafenib, and lenvatinib are included in this list, all exhibiting positive correlations with miR-3682-3p expression. Therefore, on further experimental validation of these findings, the implications for HCC drug therapy could be profound. A feasible scenario is to enhance the efficacy of these drugs through analogs or molecules similar to miR-3682-3p (17).

Before the widespread clinical application of new antitumor drugs, some experts have used metronomic capecitabine as salvage therapy after sorafenib treatment failure (59). Metronomic therapy refers to the continuous use of low-dose chemotherapy; unlike traditional chemotherapy, it does not have long periods of drug interruption, optimizing the antiangiogenic properties of the drug and reducing toxicity (60–62). Although there are now better treatment options (63), metronomic capecitabine remains a clinically significant approach for patients who cannot or do not want to use targeted or immunotherapy. This study found that the expression level of miR-3682-3p is closely related to the efficacy of capecitabine. Therefore, by measuring the expression level of miR-3682-3p, it may be possible to predict the effectiveness of capecitabine. This will guide patients with liver cancer in obtaining more rational treatment plans, making it of significant clinical importance.

### Limitations of this study

Through the method of data analysis, this article draws conclusions using publicly available data. In addition, mutual verification with other websites is conducted to enhance the credibility of the conclusions. However, it must be acknowledged that this purely bioinformatics analysis method inherently possesses limitations: namely, data can never fully depict the true situation within the human body. Therefore, the expression level of miR-3682-3p in patients with liver cancer, especially those of different ethnicities, and its role in the occurrence and progression of the disease remain unclear. This necessitates validation through biological experiments.

During the writing, submission, and revision stages of this article, the researchers noted the inclusion of several newly published articles focusing on miR-3682-3p in the database. This undoubtedly impacts the novelty of the study results. However, more researchers have observed the role of miR-3682-3p in malignant tumors, particularly liver cancer, validating the conclusions of this study. In comparison with other studies, our research uses a more systematic and comprehensive bioinformatics analysis method to derive conclusions, which is uncommon in similar articles.

This study sheds light on the multifaceted role of miR-3682-3p in HCC. We have explored its intricate connections with the tumor microenvironment, immune cell infiltration, and its potential impact on drug response. The significant associations observed between miR-3682-3p and various aspects of HCC underscore its potential as a valuable biomarker for early diagnosis, prognosis, and as a therapeutic target. The relationships with key pathways, such as complement and coagulation cascades, underline its involvement in the intricate web of HCC progression. The insights gained from our research not only provide a comprehensive view of miR-3682-3p's functions but also suggest new directions for further investigations in understanding HCC pathogenesis and treatment strategies. Our findings highlight the need for additional studies to validate these intriguing relationships and to explore innovative therapeutic approaches that harness the potential of miR-3682-3p for improving the management of HCC.

## CONFLICTS OF INTEREST

**Guarantor of the article:** Pei-Dong Miao, MMed.

**Specific author contributions:** P.-D.M. is responsible for figure creation, programming, and writing; Y.L. assists in writing; Y.-D.J. reviews. All authors approved the final manuscript.

**Financial support:** None to report.

**Potential competing interests:** None to report.

**Data availability:** The data sets used in this study are available from the corresponding author on reasonable request.

**Study Highlights**

**WHAT IS KNOWN**

✓ MiR-3682-3p has gained attention as a potential diagnostic and prognostic marker in HCC.
✓ While prior studies have touched on its significance, a comprehensive exploration of its role in liver cancer biology, encompassing expression patterns, regulatory targets, and interactions within the tumor microenvironment, is still needed.

**WHAT IS NEW HERE**

✓ This study takes an approach to investigate miR-3682-3p in liver cancer.
✓ Leveraging bioinformatics analyses, survival assessments, and functional investigations, our aim was to uncover its diagnostic utility, prognostic value, and underlying mechanisms.
✓ We also explored its relationships with immune cell presence, therapy responses, and its influence on critical signaling pathways in HCC.

## Supplementary Material
