# Supplementary material for: Celestial Insights: Unraveling the Role of miR-3682-3p in Hepatocellular Carcinoma
Source: Clin Transl Gastroenterol. 2024 Apr 25;15(4):e00690. doi: 10.14309/ctg.0000000000000690 (PMC11042776; doi:10.14309/ctg.0000000000000690)
Supplement: Supplementary file 1 [file ct9-15-e00690-s001.docx]

Table S1. 207 differential expressed miRNAs between tumor and control groups

| **miRNA** | **conMean** | **treatMean** | **logFC** | **pValue** | **fdr** |
| --- | --- | --- | --- | --- | --- |
| hsa-miR-5187-5p | 0.402 | 0.815 | 1.020 | 2.39E-04 | 4.21E-04 |
| hsa-miR-3923 | 0.074 | 19.429 | 8.039 | 1.20E-04 | 2.23E-04 |
| hsa-miR-224-3p | 2.184 | 7.033 | 1.687 | 5.58E-08 | 1.55E-07 |
| hsa-miR-4536-3p | 0.485 | 0.185 | -1.390 | 6.52E-12 | 3.30E-11 |
| hsa-miR-130a-3p | 119.932 | 54.026 | -1.151 | 2.65E-19 | 4.95E-18 |
| hsa-miR-96-5p | 1.200 | 14.461 | 3.591 | 3.28E-11 | 1.50E-10 |
| hsa-miR-765 | 0.127 | 0.465 | 1.868 | 2.75E-07 | 7.23E-07 |
| hsa-miR-10b-5p | 2034.149 | 21097.683 | 3.375 | 3.86E-19 | 6.11E-18 |
| hsa-miR-7846-3p | 0.310 | 0.146 | -1.086 | 7.03E-06 | 1.57E-05 |
| hsa-miR-10a-5p | 29472.960 | 14123.180 | -1.061 | 3.09E-17 | 3.28E-16 |
| hsa-miR-376a-2-5p | 0.931 | 0.315 | -1.561 | 6.23E-15 | 5.05E-14 |
| hsa-miR-497-5p | 36.314 | 17.036 | -1.092 | 3.38E-19 | 5.63E-18 |
| hsa-miR-7974 | 0.057 | 0.399 | 2.798 | 1.47E-05 | 3.15E-05 |
| hsa-miR-937-3p | 1.139 | 3.293 | 1.531 | 3.66E-05 | 7.34E-05 |
| hsa-miR-4685-3p | 0.320 | 0.153 | -1.069 | 5.81E-05 | 1.13E-04 |
| hsa-miR-452-5p | 60.435 | 289.822 | 2.262 | 8.64E-14 | 6.06E-13 |
| hsa-miR-1270 | 0.578 | 2.580 | 2.158 | 1.19E-04 | 2.22E-04 |
| hsa-miR-338-3p | 280.643 | 681.943 | 1.281 | 6.42E-03 | 9.26E-03 |
| hsa-miR-6516-5p | 0.165 | 0.390 | 1.244 | 1.16E-04 | 2.17E-04 |
| hsa-miR-760 | 0.202 | 0.635 | 1.653 | 5.60E-05 | 1.10E-04 |
| hsa-miR-1269a | 14.843 | 694.940 | 5.549 | 7.85E-08 | 2.16E-07 |
| hsa-miR-500b-3p | 0.988 | 2.266 | 1.197 | 7.87E-10 | 3.04E-09 |
| hsa-miR-3680-3p | 0.100 | 0.318 | 1.663 | 2.20E-04 | 3.89E-04 |
| hsa-miR-514a-3p | 3.037 | 11.068 | 1.866 | 5.50E-08 | 1.54E-07 |
| hsa-miR-33b-5p | 24.720 | 7.228 | -1.774 | 3.24E-20 | 7.41E-19 |
| hsa-miR-500b-5p | 3.154 | 6.486 | 1.040 | 2.20E-09 | 8.22E-09 |
| hsa-miR-589-5p | 42.020 | 108.369 | 1.367 | 1.76E-22 | 7.76E-21 |
| hsa-miR-133b | 0.880 | 0.381 | -1.207 | 4.26E-13 | 2.57E-12 |
| hsa-miR-135a-5p | 0.493 | 6.422 | 3.703 | 4.06E-06 | 9.32E-06 |
| hsa-miR-2114-5p | 0.319 | 2.494 | 2.965 | 1.25E-03 | 1.98E-03 |
| hsa-miR-378i | 0.330 | 0.146 | -1.179 | 2.31E-05 | 4.81E-05 |
| hsa-miR-219b-3p | 0.167 | 0.484 | 1.533 | 1.56E-06 | 3.71E-06 |
| hsa-miR-33b-3p | 1.863 | 0.636 | -1.550 | 5.96E-17 | 5.93E-16 |
| hsa-miR-26b-3p | 59.612 | 27.644 | -1.109 | 3.66E-23 | 2.51E-21 |
| hsa-miR-4742-3p | 0.459 | 1.045 | 1.185 | 6.15E-07 | 1.52E-06 |
| hsa-miR-6734-5p | 0.099 | 0.234 | 1.246 | 6.45E-04 | 1.06E-03 |
| hsa-miR-378d | 3.639 | 1.718 | -1.083 | 2.12E-14 | 1.63E-13 |
| hsa-miR-4443 | 0.686 | 0.331 | -1.052 | 5.33E-08 | 1.50E-07 |
| hsa-miR-4442 | 0.389 | 0.156 | -1.319 | 4.58E-08 | 1.30E-07 |
| hsa-miR-6788-3p | 0.236 | 0.522 | 1.148 | 3.02E-04 | 5.20E-04 |
| hsa-miR-378c | 38.227 | 18.896 | -1.016 | 4.09E-18 | 5.61E-17 |
| hsa-miR-150-3p | 2.780 | 1.239 | -1.166 | 6.31E-09 | 2.13E-08 |
| hsa-miR-130a-5p | 1.292 | 0.549 | -1.236 | 1.77E-13 | 1.19E-12 |
| hsa-miR-503-3p | 1.742 | 0.792 | -1.137 | 1.72E-11 | 8.22E-11 |
| hsa-miR-221-3p | 56.110 | 143.237 | 1.352 | 5.62E-14 | 4.13E-13 |
| hsa-miR-3691-5p | 0.196 | 0.471 | 1.262 | 1.23E-03 | 1.95E-03 |
| hsa-miR-939-5p | 0.479 | 1.018 | 1.087 | 9.83E-07 | 2.38E-06 |
| hsa-miR-3144-3p | 0.036 | 0.768 | 4.398 | 1.37E-09 | 5.25E-09 |
| hsa-miR-502-5p | 0.806 | 1.824 | 1.178 | 3.13E-06 | 7.26E-06 |
| hsa-miR-542-5p | 4.507 | 1.781 | -1.340 | 3.26E-19 | 5.58E-18 |
| hsa-miR-19a-3p | 25.580 | 69.780 | 1.448 | 7.81E-09 | 2.59E-08 |
| hsa-miR-99a-3p | 7.599 | 3.453 | -1.138 | 5.84E-18 | 7.66E-17 |
| hsa-miR-376c-5p | 1.891 | 0.941 | -1.008 | 8.64E-13 | 4.89E-12 |
| hsa-miR-636 | 0.082 | 0.234 | 1.507 | 1.07E-03 | 1.71E-03 |
| hsa-miR-643 | 0.144 | 0.449 | 1.643 | 3.94E-07 | 1.00E-06 |
| hsa-miR-511-5p | 33.613 | 12.946 | -1.376 | 8.36E-22 | 2.86E-20 |
| hsa-miR-122b-5p | 0.639 | 3.206 | 2.328 | 4.00E-07 | 1.02E-06 |
| hsa-miR-145-5p | 2310.461 | 1082.841 | -1.093 | 1.27E-17 | 1.54E-16 |
| hsa-let-7c-3p | 28.168 | 9.780 | -1.526 | 1.00E-20 | 2.48E-19 |
| hsa-miR-7-5p | 0.427 | 0.907 | 1.086 | 3.21E-02 | 4.23E-02 |
| hsa-miR-122-3p | 416.305 | 170.595 | -1.287 | 1.10E-22 | 5.21E-21 |
| hsa-miR-520a-3p | 0.210 | 48.085 | 7.837 | 3.67E-04 | 6.20E-04 |
| hsa-miR-4746-5p | 0.761 | 4.077 | 2.421 | 2.37E-19 | 4.56E-18 |
| hsa-miR-190b-5p | 0.150 | 1.317 | 3.135 | 8.64E-11 | 3.75E-10 |
| hsa-miR-6502-5p | 0.708 | 0.263 | -1.428 | 3.78E-11 | 1.70E-10 |
| hsa-miR-29c-3p | 4369.143 | 1899.391 | -1.202 | 1.10E-21 | 3.47E-20 |
| hsa-miR-4686 | 0.923 | 0.051 | -4.187 | 3.50E-51 | 2.16E-48 |
| hsa-miR-1276 | 0.100 | 0.361 | 1.846 | 9.50E-06 | 2.06E-05 |
| hsa-miR-500a-3p | 161.257 | 437.396 | 1.440 | 5.20E-17 | 5.26E-16 |
| hsa-miR-17-5p | 447.869 | 926.954 | 1.049 | 3.10E-06 | 7.21E-06 |
| hsa-miR-184 | 0.828 | 21.638 | 4.708 | 1.77E-05 | 3.75E-05 |
| hsa-miR-508-3p | 5.691 | 19.988 | 1.812 | 1.21E-08 | 3.82E-08 |
| hsa-miR-5586-5p | 1.697 | 3.769 | 1.151 | 3.31E-05 | 6.70E-05 |
| hsa-miR-509-3p | 1.378 | 5.990 | 2.120 | 1.67E-06 | 3.97E-06 |
| hsa-miR-301b-3p | 0.440 | 1.443 | 1.714 | 4.68E-07 | 1.18E-06 |
| hsa-miR-378a-5p | 302.262 | 138.075 | -1.130 | 2.89E-19 | 5.10E-18 |
| hsa-miR-125b-2-3p | 55.576 | 24.049 | -1.208 | 1.12E-21 | 3.47E-20 |
| hsa-miR-21-5p | 81595.404 | 240271.722 | 1.558 | 5.22E-23 | 3.22E-21 |
| hsa-miR-660-5p | 43.436 | 93.180 | 1.101 | 8.02E-14 | 5.69E-13 |
| hsa-miR-511-3p | 1.660 | 0.814 | -1.027 | 3.86E-13 | 2.36E-12 |
| hsa-miR-424-3p | 9.171 | 3.154 | -1.540 | 1.02E-22 | 5.21E-21 |
| hsa-miR-139-3p | 102.759 | 23.330 | -2.139 | 5.61E-29 | 8.66E-27 |
| hsa-miR-6516-3p | 0.108 | 0.261 | 1.272 | 1.04E-03 | 1.68E-03 |
| hsa-miR-34c-5p | 0.176 | 3.070 | 4.123 | 1.49E-12 | 8.04E-12 |
| hsa-miR-1226-3p | 0.526 | 1.665 | 1.663 | 1.93E-05 | 4.05E-05 |
| hsa-miR-6844 | 0.056 | 0.319 | 2.508 | 4.14E-05 | 8.21E-05 |
| hsa-miR-24-1-5p | 41.676 | 19.249 | -1.114 | 1.89E-22 | 7.78E-21 |
| hsa-miR-105-5p | 0.064 | 51.815 | 9.668 | 7.81E-11 | 3.42E-10 |
| hsa-miR-541-3p | 0.232 | 3.527 | 3.927 | 3.56E-02 | 4.59E-02 |
| hsa-miR-99a-5p | 1648.254 | 811.663 | -1.022 | 1.88E-17 | 2.11E-16 |
| hsa-miR-3934-5p | 0.106 | 0.308 | 1.535 | 1.20E-02 | 1.69E-02 |
| hsa-miR-125b-1-3p | 0.933 | 0.441 | -1.080 | 1.63E-08 | 4.97E-08 |
| hsa-miR-139-5p | 377.320 | 118.185 | -1.675 | 2.14E-25 | 2.20E-23 |
| hsa-miR-6514-5p | 0.109 | 0.299 | 1.459 | 2.01E-05 | 4.22E-05 |
| hsa-miR-34c-3p | 0.243 | 1.811 | 2.900 | 2.53E-09 | 9.31E-09 |
| hsa-miR-3127-5p | 1.616 | 3.473 | 1.104 | 1.56E-08 | 4.84E-08 |
| hsa-miR-512-3p | 0.124 | 33.822 | 8.093 | 4.84E-04 | 8.10E-04 |
| hsa-miR-9-5p | 133.773 | 1363.768 | 3.350 | 7.98E-05 | 1.53E-04 |
| hsa-miR-10b-3p | 0.881 | 8.945 | 3.344 | 8.93E-18 | 1.12E-16 |
| hsa-miR-6503-5p | 0.881 | 0.280 | -1.653 | 5.18E-13 | 3.01E-12 |
| hsa-miR-501-5p | 1.640 | 4.159 | 1.342 | 4.57E-10 | 1.84E-09 |
| hsa-miR-18a-5p | 8.496 | 26.267 | 1.628 | 5.91E-06 | 1.33E-05 |
| hsa-miR-25-5p | 0.109 | 0.309 | 1.498 | 6.23E-04 | 1.02E-03 |
| hsa-miR-1266-5p | 2.285 | 8.110 | 1.827 | 4.64E-13 | 2.73E-12 |
| hsa-miR-5590-5p | 0.341 | 0.166 | -1.040 | 5.23E-06 | 1.19E-05 |
| hsa-miR-4661-5p | 2.317 | 8.181 | 1.820 | 4.01E-09 | 1.41E-08 |
| hsa-miR-4454 | 0.401 | 0.176 | -1.185 | 5.16E-07 | 1.29E-06 |
| hsa-miR-424-5p | 648.262 | 119.679 | -2.437 | 1.88E-27 | 2.32E-25 |
| hsa-miR-182-5p | 1177.427 | 10500.246 | 3.157 | 2.59E-10 | 1.08E-09 |
| hsa-miR-6783-3p | 0.054 | 0.271 | 2.334 | 3.29E-05 | 6.67E-05 |
| hsa-miR-1275 | 0.620 | 0.288 | -1.105 | 4.33E-08 | 1.24E-07 |
| hsa-let-7c-5p | 5606.981 | 2227.942 | -1.332 | 2.08E-23 | 1.60E-21 |
| hsa-miR-1180-3p | 9.030 | 27.639 | 1.614 | 3.09E-12 | 1.61E-11 |
| hsa-miR-3200-3p | 0.878 | 4.950 | 2.495 | 3.56E-08 | 1.03E-07 |
| hsa-miR-1248 | 1.431 | 0.324 | -2.142 | 1.19E-17 | 1.47E-16 |
| hsa-miR-101-3p | 48257.300 | 19689.722 | -1.293 | 1.33E-24 | 1.17E-22 |
| hsa-miR-451a | 1900.605 | 892.532 | -1.090 | 1.86E-17 | 2.11E-16 |
| hsa-miR-34a-3p | 0.586 | 1.197 | 1.030 | 2.19E-07 | 5.86E-07 |
| hsa-miR-455-3p | 1541.147 | 755.050 | -1.029 | 1.67E-18 | 2.39E-17 |
| hsa-miR-3662 | 0.139 | 1.065 | 2.937 | 3.71E-09 | 1.32E-08 |
| hsa-miR-7850-5p | 0.316 | 0.146 | -1.112 | 2.21E-09 | 8.22E-09 |
| hsa-miR-130b-3p | 15.480 | 32.442 | 1.067 | 3.97E-05 | 7.90E-05 |
| hsa-miR-301b-5p | 0.158 | 0.499 | 1.658 | 3.13E-03 | 4.70E-03 |
| hsa-miR-942-3p | 0.209 | 0.520 | 1.311 | 1.71E-04 | 3.12E-04 |
| hsa-miR-34b-3p | 0.042 | 0.641 | 3.933 | 3.91E-07 | 1.00E-06 |
| hsa-miR-214-3p | 9.251 | 3.069 | -1.592 | 4.87E-18 | 6.53E-17 |
| hsa-miR-195-5p | 82.158 | 29.394 | -1.483 | 4.44E-22 | 1.61E-20 |
| hsa-miR-891a-5p | 0.198 | 16.763 | 6.405 | 1.56E-10 | 6.58E-10 |
| hsa-miR-18a-3p | 0.941 | 2.130 | 1.178 | 4.55E-03 | 6.70E-03 |
| hsa-miR-5589-5p | 28.193 | 10.153 | -1.473 | 2.18E-19 | 4.33E-18 |
| hsa-miR-5003-3p | 0.133 | 0.420 | 1.660 | 1.85E-06 | 4.33E-06 |
| hsa-miR-7706 | 0.715 | 2.617 | 1.872 | 8.81E-18 | 1.12E-16 |
| hsa-miR-767-5p | 0.033 | 34.141 | 10.030 | 3.54E-11 | 1.60E-10 |
| hsa-miR-1258 | 14.940 | 1.302 | -3.521 | 8.29E-30 | 1.71E-27 |
| hsa-miR-224-5p | 22.534 | 196.295 | 3.123 | 4.10E-16 | 3.67E-15 |
| hsa-miR-500a-5p | 3.154 | 6.484 | 1.040 | 2.29E-09 | 8.45E-09 |
| hsa-miR-589-3p | 0.817 | 2.082 | 1.349 | 1.83E-10 | 7.68E-10 |
| hsa-miR-222-3p | 13.782 | 34.083 | 1.306 | 3.84E-12 | 1.99E-11 |
| hsa-miR-452-3p | 2.673 | 12.581 | 2.235 | 2.76E-11 | 1.28E-10 |
| hsa-miR-542-3p | 534.513 | 249.435 | -1.100 | 1.08E-19 | 2.22E-18 |
| hsa-miR-501-3p | 28.877 | 71.511 | 1.308 | 2.06E-14 | 1.63E-13 |
| hsa-miR-1269b | 1.436 | 113.901 | 6.310 | 2.33E-07 | 6.14E-07 |
| hsa-miR-103a-2-5p | 0.696 | 1.727 | 1.310 | 4.59E-09 | 1.59E-08 |
| hsa-miR-369-5p | 13.892 | 4.713 | -1.560 | 3.84E-19 | 6.11E-18 |
| hsa-miR-144-3p | 56.539 | 14.605 | -1.953 | 1.05E-22 | 5.21E-21 |
| hsa-miR-214-5p | 25.546 | 11.958 | -1.095 | 9.06E-13 | 5.06E-12 |
| hsa-miR-450a-1-3p | 0.212 | 0.102 | -1.054 | 8.09E-05 | 1.54E-04 |
| hsa-miR-3117-3p | 0.136 | 0.590 | 2.119 | 1.70E-03 | 2.66E-03 |
| hsa-miR-526b-5p | 0.619 | 69.516 | 6.812 | 3.43E-03 | 5.14E-03 |
| hsa-miR-199b-3p | 2669.845 | 970.016 | -1.461 | 1.54E-18 | 2.32E-17 |
| hsa-miR-671-5p | 1.801 | 3.749 | 1.058 | 4.10E-09 | 1.44E-08 |
| hsa-miR-146b-3p | 359.721 | 165.754 | -1.118 | 9.23E-09 | 2.97E-08 |
| hsa-miR-4664-5p | 0.164 | 0.448 | 1.450 | 1.37E-04 | 2.55E-04 |
| hsa-miR-20b-5p | 8.983 | 22.316 | 1.313 | 2.49E-02 | 3.34E-02 |
| hsa-miR-3614-5p | 6.685 | 2.468 | -1.438 | 9.01E-17 | 8.56E-16 |
| hsa-miR-551a | 0.462 | 0.176 | -1.396 | 7.37E-10 | 2.86E-09 |
| hsa-miR-6716-3p | 0.167 | 0.386 | 1.207 | 3.40E-04 | 5.78E-04 |
| hsa-miR-548d-3p | 0.111 | 0.412 | 1.900 | 7.40E-06 | 1.64E-05 |
| hsa-miR-877-5p | 0.659 | 2.250 | 1.771 | 2.93E-12 | 1.54E-11 |
| hsa-miR-142-3p | 3090.397 | 1541.740 | -1.003 | 2.50E-15 | 2.14E-14 |
| hsa-miR-552-5p | 1.413 | 41.752 | 4.885 | 4.14E-02 | 5.29E-02 |
| hsa-miR-1229-3p | 0.286 | 0.748 | 1.385 | 1.55E-04 | 2.85E-04 |
| hsa-miR-4683 | 0.379 | 0.121 | -1.644 | 9.62E-09 | 3.07E-08 |
| hsa-miR-2114-3p | 0.140 | 0.929 | 2.731 | 2.07E-02 | 2.79E-02 |
| hsa-miR-3677-5p | 0.529 | 1.201 | 1.182 | 3.55E-05 | 7.15E-05 |
| hsa-miR-454-5p | 0.293 | 0.614 | 1.070 | 1.56E-05 | 3.32E-05 |
| hsa-miR-3682-3p | 0.878 | 1.782 | 1.021 | 1.62E-05 | 3.45E-05 |
| hsa-miR-4800-3p | 0.347 | 0.076 | -2.183 | 1.86E-13 | 1.22E-12 |
| hsa-miR-142-5p | 130.918 | 61.397 | -1.092 | 1.58E-13 | 1.08E-12 |
| hsa-miR-450a-5p | 22.681 | 8.967 | -1.339 | 2.08E-21 | 6.11E-20 |
| hsa-miR-4326 | 2.233 | 6.564 | 1.556 | 2.41E-08 | 7.15E-08 |
| hsa-miR-183-5p | 318.268 | 4117.438 | 3.693 | 3.13E-11 | 1.44E-10 |
| hsa-miR-766-5p | 0.124 | 0.260 | 1.068 | 2.03E-02 | 2.74E-02 |
| hsa-miR-1294 | 0.281 | 0.128 | -1.138 | 3.26E-05 | 6.64E-05 |
| hsa-miR-337-5p | 0.750 | 0.286 | -1.389 | 7.24E-12 | 3.60E-11 |
| hsa-miR-188-5p | 1.496 | 3.938 | 1.397 | 4.37E-12 | 2.25E-11 |
| hsa-miR-205-5p | 0.644 | 10.160 | 3.979 | 2.52E-03 | 3.84E-03 |
| hsa-miR-3677-3p | 1.459 | 4.312 | 1.563 | 2.91E-09 | 1.06E-08 |
| hsa-miR-199a-3p | 2675.146 | 974.138 | -1.457 | 1.61E-18 | 2.37E-17 |
| hsa-miR-20a-5p | 365.620 | 798.314 | 1.127 | 5.58E-08 | 1.55E-07 |
| hsa-miR-581 | 0.305 | 0.788 | 1.369 | 1.57E-09 | 5.95E-09 |
| hsa-miR-1307-3p | 862.070 | 1751.219 | 1.022 | 2.10E-11 | 9.88E-11 |
| hsa-miR-326 | 11.208 | 4.278 | -1.390 | 5.82E-21 | 1.50E-19 |
| hsa-miR-5589-3p | 18.630 | 7.479 | -1.317 | 3.57E-16 | 3.29E-15 |
| hsa-miR-183-3p | 0.045 | 0.396 | 3.146 | 2.90E-07 | 7.54E-07 |
| hsa-miR-376b-5p | 1.825 | 0.847 | -1.107 | 3.60E-13 | 2.25E-12 |
| hsa-miR-125b-5p | 1201.988 | 570.095 | -1.076 | 3.25E-21 | 8.71E-20 |
| hsa-miR-4791 | 0.748 | 0.316 | -1.243 | 1.60E-09 | 6.01E-09 |
| hsa-miR-490-3p | 8.665 | 0.793 | -3.450 | 6.99E-31 | 2.16E-28 |
| hsa-miR-30c-1-3p | 3.509 | 1.533 | -1.195 | 1.80E-17 | 2.10E-16 |
| hsa-miR-93-5p | 2313.601 | 6753.493 | 1.545 | 2.66E-21 | 7.46E-20 |
| hsa-miR-147b-3p | 0.149 | 0.424 | 1.510 | 3.78E-02 | 4.86E-02 |
| hsa-miR-577 | 0.236 | 1.320 | 2.486 | 3.29E-02 | 4.31E-02 |
| hsa-miR-3614-3p | 2.635 | 1.276 | -1.046 | 6.60E-10 | 2.60E-09 |
| hsa-miR-532-5p | 702.638 | 1618.602 | 1.204 | 2.64E-17 | 2.85E-16 |
| hsa-miR-1292-5p | 0.244 | 0.539 | 1.146 | 1.45E-05 | 3.12E-05 |
| hsa-miR-10a-3p | 3.077 | 1.219 | -1.336 | 1.27E-15 | 1.10E-14 |
| hsa-miR-30a-3p | 9412.735 | 4427.968 | -1.088 | 9.75E-20 | 2.08E-18 |
| hsa-miR-335-5p | 24.931 | 9.920 | -1.329 | 1.33E-17 | 1.57E-16 |
| hsa-miR-301a-3p | 3.324 | 8.116 | 1.288 | 1.71E-08 | 5.18E-08 |
| hsa-miR-1251-5p | 0.043 | 2.224 | 5.708 | 1.13E-07 | 3.07E-07 |
| hsa-miR-1301-3p | 4.350 | 11.849 | 1.446 | 9.72E-12 | 4.80E-11 |
| hsa-miR-4664-3p | 0.074 | 0.506 | 2.764 | 1.14E-08 | 3.63E-08 |
| hsa-miR-30e-3p | 15740.457 | 7595.481 | -1.051 | 2.32E-22 | 8.96E-21 |
| hsa-miR-421 | 1.496 | 3.711 | 1.311 | 3.33E-09 | 1.20E-08 |
| hsa-miR-34a-5p | 177.127 | 384.172 | 1.117 | 4.42E-13 | 2.65E-12 |
| hsa-miR-4751 | 0.244 | 0.107 | -1.190 | 2.61E-08 | 7.63E-08 |

Table S2. 7 miRNAs associated with the prognosis of liver cancer patients

| **miRNA** | **HR** | **HR.95L** | **HR.95H** | **KMpvalue** | **COXpvalue** |
| --- | --- | --- | --- | --- | --- |
| hsa-miR-7-5p | 1.190715 | 1.086139 | 1.305360 | 1.35E-02 | 1.98E-04 |
| hsa-miR-139-5p | 0.997579 | 0.995228 | 0.999936 | 2.93E-03 | 4.41E-02 |
| hsa-miR-9-5p | 1.000060 | 1.000025 | 1.000094 | 2.47E-02 | 6.97E-04 |
| hsa-miR-4661-5p | 1.027593 | 1.013324 | 1.042063 | 5.46E-03 | 1.36E-04 |
| hsa-miR-551a | 2.822022 | 1.419198 | 5.611484 | 4.56E-02 | 3.09E-03 |
| hsa-miR-3682-3p | 1.201613 | 1.114202 | 1.295881 | 2.65E-02 | 1.88E-06 |
| hsa-miR-3677-3p | 1.063672 | 1.026101 | 1.102619 | 2.81E-02 | 7.68E-04 |

Table S3. 5 miRNAs as independent prognostic factors for liver cancer patients

| **id** | **HR** | **HR.95L** | **HR.95H** | **pvalue** |
| --- | --- | --- | --- | --- |
| hsa-miR-7-5p | 1.171859672 | 1.055597978 | 1.300926223 | 0.002930525 |
| hsa-miR-9-5p | 1.000062709 | 1.000020372 | 1.000105047 | 0.003694523 |
| hsa-miR-4661-5p | 1.032778493 | 1.016503987 | 1.049313558 | 6.90E-05 |
| hsa-miR-3682-3p | 1.221240655 | 1.120731744 | 1.330763357 | 5.09E-06 |
| hsa-miR-3677-3p | 1.048819224 | 1.003655182 | 1.096015628 | 0.033802056 |

Table S4. miRNAs predict liver cancer prognosis with an AUC>0.6

| **miRNA** | **AUC** |
| --- | --- |
| hsa-miR-9-5p | 0.66969916404661 |
| hsa-miR-4661-5p | 0.619721524373859 |
| hsa-miR-3682-3p | 0.613544066931614 |
| hsa-miR-3677-3p | 0.61601722211728 |

Table S5. 51 target genes predicted by the TargetScan website to be directly regulated by miR-3682-3p

| **miRNA** | **Gene** | **cor** | **pvalue** |
| --- | --- | --- | --- |
| hsa-miR-3682-3p | CFHR4 | -0.196399988 | 0.000143451 |
| hsa-miR-3682-3p | CYYR1 | -0.184127774 | 0.000370472 |
| hsa-miR-3682-3p | PLA2G5 | -0.196523276 | 0.000142048 |
| hsa-miR-3682-3p | TMEM154 | -0.12323825 | 0.017713127 |
| hsa-miR-3682-3p | CXCL12 | -0.141213524 | 0.00651336 |
| hsa-miR-3682-3p | HOPX | -0.14135159 | 0.006460611 |
| hsa-miR-3682-3p | TPRG1 | -0.165076077 | 0.001440326 |
| hsa-miR-3682-3p | CYP2E1 | -0.149352255 | 0.003985677 |
| hsa-miR-3682-3p | TACR1 | -0.104140286 | 0.045301247 |
| hsa-miR-3682-3p | TIGD2 | -0.158394838 | 0.002244688 |
| hsa-miR-3682-3p | GABARAPL1 | -0.170844885 | 0.000968789 |
| hsa-miR-3682-3p | ITPRIP | -0.118209969 | 0.022960027 |
| hsa-miR-3682-3p | TIAM1 | -0.116846866 | 0.024595717 |
| hsa-miR-3682-3p | NFASC | -0.109757523 | 0.034818371 |
| hsa-miR-3682-3p | EXPH5 | -0.146790203 | 0.004664213 |
| hsa-miR-3682-3p | SLIT3 | -0.183163388 | 0.000398159 |
| hsa-miR-3682-3p | ENDOD1 | -0.169299495 | 0.001078705 |
| hsa-miR-3682-3p | MMAA | -0.216054292 | 2.77E-05 |
| hsa-miR-3682-3p | GALNT15 | -0.14517081 | 0.005145142 |
| hsa-miR-3682-3p | XYLT1 | -0.121116781 | 0.019783432 |
| hsa-miR-3682-3p | ANKRD29 | -0.122582454 | 0.018331927 |
| hsa-miR-3682-3p | NOVA2 | -0.150894924 | 0.003621499 |
| hsa-miR-3682-3p | C1RL | -0.153957862 | 0.002986347 |
| hsa-miR-3682-3p | ITGA11 | -0.129662591 | 0.012552589 |
| hsa-miR-3682-3p | SLC14A1 | -0.147295971 | 0.004522542 |
| hsa-miR-3682-3p | TCF21 | -0.196097754 | 0.000146947 |
| hsa-miR-3682-3p | RBMS3 | -0.159750183 | 0.002054239 |
| hsa-miR-3682-3p | MICAL2 | -0.110519073 | 0.033570104 |
| hsa-miR-3682-3p | PLAT | -0.191091448 | 0.000217812 |
| hsa-miR-3682-3p | CDKN1A | -0.115819609 | 0.025893946 |
| hsa-miR-3682-3p | TOX | -0.134357417 | 0.009669948 |
| hsa-miR-3682-3p | BST1 | -0.109348249 | 0.035505377 |
| hsa-miR-3682-3p | OCIAD2 | -0.179837461 | 0.000509108 |
| hsa-miR-3682-3p | CDC37L1 | -0.157889728 | 0.002319692 |
| hsa-miR-3682-3p | SOD2 | -0.102465352 | 0.048898235 |
| hsa-miR-3682-3p | CHST7 | -0.12987055 | 0.012410412 |
| hsa-miR-3682-3p | CLYBL | -0.119921915 | 0.021039705 |
| hsa-miR-3682-3p | ECHDC2 | -0.153195724 | 0.003134145 |
| hsa-miR-3682-3p | GNG11 | -0.148165943 | 0.00428791 |
| hsa-miR-3682-3p | BNC2 | -0.107974795 | 0.03789563 |
| hsa-miR-3682-3p | SERTAD1 | -0.112041309 | 0.031188938 |
| hsa-miR-3682-3p | C3 | -0.112740989 | 0.030143822 |
| hsa-miR-3682-3p | FMO3 | -0.122484979 | 0.018425495 |
| hsa-miR-3682-3p | C7 | -0.20499714 | 7.12E-05 |
| hsa-miR-3682-3p | WLS | -0.211174339 | 4.23E-05 |
| hsa-miR-3682-3p | GLYAT | -0.208985389 | 5.10E-05 |
| hsa-miR-3682-3p | TTPA | -0.104713749 | 0.044121915 |
| hsa-miR-3682-3p | CLEC14A | -0.137458502 | 0.008104142 |
| hsa-miR-3682-3p | BMP10 | -0.178138104 | 0.00057629 |
| hsa-miR-3682-3p | PLLP | -0.132540081 | 0.010707531 |
| hsa-miR-3682-3p | LUM | -0.183052264 | 0.00040147 |

Table S6. GSEA results of different expressed genes between high and low expression groups based on the median hsa-miR-3682-3p expression level

| **ID** | **setSize** | **enrichmentScore** | **NES** | **pvalue** |
| --- | --- | --- | --- | --- |
| KEGG_CELL_CYCLE | 121 | 0.563569318 | 2.089870499 | 2.13E-06 |
| KEGG_HEDGEHOG_SIGNALING_PATHWAY | 50 | -0.588154268 | -2.176338008 | 0.000202207 |
| KEGG_COMPLEMENT_AND_COAGULATION_CASCADES | 69 | -0.525701595 | -2.101653504 | 0.000333507 |
| KEGG_O_GLYCAN_BIOSYNTHESIS | 24 | -0.661747899 | -2.110452378 | 0.000580848 |
| KEGG_BASAL_CELL_CARCINOMA | 52 | -0.555074583 | -2.06183101 | 0.000894524 |
| KEGG_DNA_REPLICATION | 34 | 0.623626295 | 1.881901793 | 0.001600675 |
| KEGG_RENIN_ANGIOTENSIN_SYSTEM | 15 | -0.710168548 | -2.023708315 | 0.003003097 |
| KEGG_TASTE_TRANSDUCTION | 19 | -0.645322518 | -1.93506297 | 0.003101841 |
| KEGG_ECM_RECEPTOR_INTERACTION | 75 | -0.452669466 | -1.824213039 | 0.002723846 |
| KEGG_MELANOGENESIS | 91 | -0.426895623 | -1.785088409 | 0.002571535 |
| KEGG_STARCH_AND_SUCROSE_METABOLISM | 44 | 0.55341492 | 1.761484481 | 0.002191871 |
| KEGG_FOCAL_ADHESION | 183 | -0.327593326 | -1.527704683 | 0.004260788 |
| KEGG_CALCIUM_SIGNALING_PATHWAY | 132 | -0.366333764 | -1.631301645 | 0.00557217 |
| KEGG_GRAFT_VERSUS_HOST_DISEASE | 31 | -0.561965482 | -1.875427364 | 0.006825895 |
| KEGG_TRYPTOPHAN_METABOLISM | 36 | -0.500384985 | -1.729781698 | 0.006986555 |
| KEGG_ASCORBATE_AND_ALDARATE_METABOLISM | 24 | 0.61914123 | 1.748001884 | 0.008264835 |
| KEGG_PPAR_SIGNALING_PATHWAY | 62 | -0.427936516 | -1.667726078 | 0.008349532 |
| KEGG_HEMATOPOIETIC_CELL_LINEAGE | 69 | 0.477045731 | 1.648881274 | 0.010740629 |
| KEGG_PENTOSE_AND_GLUCURONATE_INTERCONVERSIONS | 26 | 0.581511088 | 1.669757198 | 0.012292253 |
| KEGG_OOCYTE_MEIOSIS | 100 | 0.434351603 | 1.564775433 | 0.012750667 |
| KEGG_AUTOIMMUNE_THYROID_DISEASE | 31 | -0.532766305 | -1.777981992 | 0.013719204 |
| KEGG_LINOLEIC_ACID_METABOLISM | 24 | -0.543086135 | -1.732015209 | 0.018545975 |
| KEGG_ALDOSTERONE_REGULATED_SODIUM_REABSORPTION | 33 | -0.479207456 | -1.625385682 | 0.019144577 |
| KEGG_WNT_SIGNALING_PATHWAY | 142 | -0.314339121 | -1.409261448 | 0.021178762 |
| KEGG_MATURITY_ONSET_DIABETES_OF_THE_YOUNG | 18 | 0.646216329 | 1.737447836 | 0.022487874 |
| KEGG_VASCULAR_SMOOTH_MUSCLE_CONTRACTION | 99 | -0.345812224 | -1.465263147 | 0.023328146 |
| KEGG_DILATED_CARDIOMYOPATHY | 70 | -0.392142966 | -1.566504937 | 0.026139223 |
| KEGG_PRIMARY_BILE_ACID_BIOSYNTHESIS | 16 | -0.569244713 | -1.674219667 | 0.029691424 |
| KEGG_SPLICEOSOME | 125 | 0.390895506 | 1.457715008 | 0.032258065 |
| KEGG_PROGESTERONE_MEDIATED_OOCYTE_MATURATION | 75 | 0.424745488 | 1.48071051 | 0.035220126 |
| KEGG_HOMOLOGOUS_RECOMBINATION | 26 | 0.547792156 | 1.572936293 | 0.037921348 |
| KEGG_PATHWAYS_IN_CANCER | 305 | -0.248009722 | -1.232965941 | 0.037970322 |
| KEGG_ALLOGRAFT_REJECTION | 31 | -0.473282355 | -1.579468326 | 0.042315987 |
| KEGG_DRUG_METABOLISM_CYTOCHROME_P450 | 68 | 0.425463553 | 1.47003759 | 0.046835443 |

Table S7. 91 drugs may be associated with the expression level of miR-3682-3p

| **drug** | **meanL** | **meanH** | **mean_ratio** | **Pvalue** |
| --- | --- | --- | --- | --- |
| Eg5_9814 | 1.14086578 | 0.775632581 | 0.679863131 | 0.012955081 |
| BI-2536 | 1.598898221 | 1.155431581 | 0.722642358 | 0.001178499 |
| Lapatinib | 4.672244444 | 4.285639377 | 0.917254957 | 0.002272183 |
| Fulvestrant | 4.425519144 | 4.21291172 | 0.951958761 | 0.049903786 |
| Selumetinib | 6.024668262 | 6.060037782 | 1.005870783 | 0.021044487 |
| XAV939 | 6.287485684 | 6.454448762 | 1.026554824 | 0.019199823 |
| Carmustine | 8.710038425 | 9.003391962 | 1.033679936 | 0.022805414 |
| AZD1208 | 7.501373301 | 7.7663784 | 1.035327545 | 0.003701306 |
| SB216763 | 7.351352713 | 7.658597526 | 1.041794323 | 1.87E-05 |
| Picolinici-acid | 7.292727324 | 7.597664416 | 1.041813862 | 0.004730812 |
| SB505124 | 3.361320575 | 3.510193679 | 1.044290064 | 0.044224139 |
| GSK343 | 4.072959745 | 4.255061809 | 1.044710008 | 0.020235808 |
| AZD8055 | 0.855874489 | 0.894146318 | 1.044716636 | 0.00289823 |
| GSK591 | 6.439145665 | 6.764859083 | 1.050583328 | 0.000211737 |
| PF-4708671 | 5.51125354 | 5.791458731 | 1.05084237 | 0.031266197 |
| Palbociclib | 5.169892508 | 5.439742867 | 1.052196513 | 0.005460014 |
| PAK_5339 | 3.46820069 | 3.651712867 | 1.052912791 | 0.024819278 |
| IRAK4_4710 | 6.900173988 | 7.272566751 | 1.053968605 | 0.006576751 |
| Ribociclib | 5.464185352 | 5.762454731 | 1.054586248 | 6.08E-05 |
| Doramapimod | 6.363461811 | 6.773396775 | 1.064420118 | 5.22E-06 |
| LJI308 | 7.126297322 | 7.586068042 | 1.064517477 | 1.82E-06 |
| Olaparib | 6.017377136 | 6.408458622 | 1.064992019 | 0.003335142 |
| Tozasertib | 4.182643991 | 4.45675548 | 1.065535458 | 0.025915573 |
| GSK2606414 | 5.248860503 | 5.597222925 | 1.066369152 | 0.01184276 |
| Axitinib | 4.372718176 | 4.664837099 | 1.066804882 | 0.013904605 |
| MIM1 | 5.529109435 | 5.901343588 | 1.067322624 | 0.028661503 |
| AGI-6780 | 5.862134517 | 6.260293614 | 1.067920498 | 0.0067349 |
| Cisplatin | 4.633893215 | 4.956719959 | 1.069666418 | 0.046125109 |
| AZD5363 | 4.191304073 | 4.487985707 | 1.070785042 | 0.017220029 |
| NU7441 | 3.754311397 | 4.027844945 | 1.072858514 | 0.000913578 |
| Niraparib | 5.954622953 | 6.395643489 | 1.074063554 | 0.001356803 |
| PCI-34051 | 6.228280868 | 6.696997576 | 1.075256193 | 0.043095312 |
| Ulixertinib | 3.981015273 | 4.291714451 | 1.078045211 | 0.019660137 |
| Dabrafenib | 6.282644901 | 6.773654845 | 1.078153382 | 0.000309566 |
| GSK1904529A | 6.115617311 | 6.599905823 | 1.079188819 | 0.008223376 |
| Venetoclax | 3.128485432 | 3.379207592 | 1.080141706 | 0.011387654 |
| LY2109761 | 7.20441454 | 7.804107194 | 1.08323961 | 0.010948142 |
| Oxaliplatin | 5.224367661 | 5.660412004 | 1.083463564 | 0.003759868 |
| Afuresertib | 3.634287632 | 3.938568281 | 1.083724977 | 0.026647042 |
| LGK974 | 5.709425863 | 6.194876837 | 1.085026233 | 0.019403231 |
| AZD3759 | 3.788646705 | 4.111794599 | 1.085293752 | 0.011711112 |
| ZM447439 | 4.233847159 | 4.597451321 | 1.085880323 | 0.008917424 |
| KU-55933 | 6.165442099 | 6.701400208 | 1.086929388 | 0.000108875 |
| Nelarabine | 8.049687137 | 8.763205384 | 1.088639252 | 0.001737098 |
| Mirin | 6.898924571 | 7.51312795 | 1.089028859 | 0.000239469 |
| OF-1 | 5.708664196 | 6.23007303 | 1.0913364 | 0.020288864 |
| Sinularin | 4.998770711 | 5.456016975 | 1.091471742 | 0.010171734 |
| Gallibiscoquinazole | 3.783537671 | 4.1426542 | 1.094915542 | 0.012143778 |
| Foretinib | 1.82860178 | 2.004159063 | 1.096006295 | 0.004116449 |
| PLX-4720 | 6.080318457 | 6.679618725 | 1.098563961 | 0.003442457 |
| JAK1_8709 | 5.68861271 | 6.252872408 | 1.09919109 | 0.003335142 |
| AZD1332 | 5.25405005 | 5.832178408 | 1.110034802 | 0.003109697 |
| AT13148 | 5.062639137 | 5.646464465 | 1.115320352 | 1.99E-05 |
| VE-822 | 4.621496981 | 5.177572752 | 1.120323733 | 0.004142137 |
| TAF1_5496 | 5.193272856 | 5.824277627 | 1.121504259 | 0.030502044 |
| CDK9_5576 | 1.439520552 | 1.61685641 | 1.123190918 | 0.010014428 |
| ERK_6604 | 4.588597039 | 5.253136245 | 1.14482405 | 0.003251495 |
| JAK_8517 | 4.245299119 | 4.862978447 | 1.145497245 | 0.004630094 |
| Entinostat | 3.180507543 | 3.654345397 | 1.148981836 | 5.62E-05 |
| JQ1 | 3.370603288 | 3.889783994 | 1.154031983 | 0.002141658 |
| Fludarabine | 6.269830012 | 7.282759235 | 1.16155609 | 0.000976629 |
| AZ960 | 3.04438189 | 3.549403616 | 1.165886457 | 0.001523198 |
| Irinotecan | 3.77123022 | 4.416959484 | 1.171225098 | 0.040235453 |
| Dactolisib | 0.298340576 | 0.350510654 | 1.174867525 | 0.029680058 |
| VSP34_8731 | 3.518454571 | 4.185184249 | 1.189495037 | 0.002735067 |
| AZD2014 | 2.989870847 | 3.576166286 | 1.196093902 | 9.43E-06 |
| Cytarabine | 2.567413535 | 3.079360925 | 1.199401999 | 0.001432899 |
| KRAS (G12C) Inhibitor-12 | 6.163784166 | 7.39804559 | 1.200244102 | 0.016016205 |
| AZD5153 | 2.535621779 | 3.071794059 | 1.211455937 | 0.000243351 |
| IGF1R_3801 | 2.665416656 | 3.231780305 | 1.212485972 | 0.001065737 |
| PD0325901 | 1.402511672 | 1.708048093 | 1.217849467 | 0.001467412 |
| Obatoclax Mesylate | 2.371664132 | 2.936353012 | 1.238098166 | 0.001036436 |
| BMS-754807 | 1.30160625 | 1.615939716 | 1.241496587 | 0.002219311 |
| Podophyllotoxin bromide | 1.303097144 | 1.626569775 | 1.248233704 | 0.013759076 |
| Alisertib | 2.869036063 | 3.623052766 | 1.26281186 | 0.000619029 |
| AZD6482 | 4.526414533 | 5.72496559 | 1.264790387 | 0.004789251 |
| ERK_2440 | 3.530817952 | 4.481517018 | 1.269257458 | 0.002752772 |
| Nutlin-3a (-) | 5.74909638 | 7.303264815 | 1.270332646 | 8.11E-09 |
| AZD5438 | 3.33749631 | 4.276923062 | 1.281476492 | 0.002913905 |
| Elephantin | 5.036809101 | 6.516089454 | 1.293693949 | 0.037803756 |
| Rapamycin | 0.277299329 | 0.368308798 | 1.328199386 | 0.038727666 |
| Dactinomycin | 0.318411386 | 0.43192046 | 1.356485599 | 0.019608519 |
| Vincristine | 1.921976877 | 2.637172241 | 1.372114447 | 0.03020563 |
| Sabutoclax | 1.002101479 | 1.408263253 | 1.405310024 | 0.009125108 |
| Epirubicin | 0.965867997 | 1.3736906 | 1.422234306 | 0.012279726 |
| Mitoxantrone | 1.545071179 | 2.2876024 | 1.480580592 | 4.06E-05 |
| Gemcitabine | 0.853338983 | 1.290334331 | 1.512100532 | 8.82E-05 |
| Topotecan | 1.45045654 | 2.229170383 | 1.536874991 | 0.001040057 |
| Teniposide | 1.921544936 | 2.954666534 | 1.537651542 | 0.003040933 |
| Camptothecin | 0.355446127 | 0.665141398 | 1.871286107 | 0.015716442 |
| CDK9_5038 | 0.739566678 | 1.455749787 | 1.968382069 | 0.003891827 |

Table S8. KEGG enrichment analysis of 51 genes

| **ID** | **Description** | **GeneRatio** | **BgRatio** | **pvalue** | **geneID** |
| --- | --- | --- | --- | --- | --- |
| hsa04610 | Complement and coagulation cascades | 4/23 | 86/8577 | 7.21E-05 | CFHR4/PLAT/C3/C7 |
| hsa00532 | Glycosaminoglycan biosynthesis - chondroitin sulfate / dermatan sulfate | 2/23 | 21/8577 | 1.40E-03 | XYLT1/CHST7 |
| hsa00591 | Linoleic acid metabolism | 2/23 | 30/8577 | 2.86E-03 | PLA2G5/CYP2E1 |
| hsa04810 | Regulation of actin cytoskeleton | 4/23 | 229/8577 | 2.94E-03 | CXCL12/TIAM1/ITGA11/C7 |
| hsa04068 | FoxO signaling pathway | 3/23 | 131/8577 | 4.93E-03 | GABARAPL1/CDKN1A/SOD2 |
| hsa00590 | Arachidonic acid metabolism | 2/23 | 61/8577 | 1.14E-02 | PLA2G5/CYP2E1 |
| hsa04062 | Chemokine signaling pathway | 3/23 | 192/8577 | 1.41E-02 | CXCL12/TIAM1/GNG11 |
| hsa05167 | Kaposi sarcoma-associated herpesvirus infection | 3/23 | 194/8577 | 1.45E-02 | CDKN1A/GNG11/C3 |
| hsa00982 | Drug metabolism - cytochrome P450 | 2/23 | 72/8577 | 1.57E-02 | CYP2E1/FMO3 |
| hsa05205 | Proteoglycans in cancer | 3/23 | 205/8577 | 1.68E-02 | TIAM1/CDKN1A/LUM |
| hsa05163 | Human cytomegalovirus infection | 3/23 | 225/8577 | 2.14E-02 | CXCL12/CDKN1A/GNG11 |
| hsa04727 | GABAergic synapse | 2/23 | 89/8577 | 2.34E-02 | GABARAPL1/GNG11 |
| hsa04014 | Ras signaling pathway | 3/23 | 236/8577 | 2.43E-02 | PLA2G5/TIAM1/GNG11 |
| hsa05215 | Prostate cancer | 2/23 | 97/8577 | 2.74E-02 | PLAT/CDKN1A |
| hsa04972 | Pancreatic secretion | 2/23 | 102/8577 | 3.01E-02 | PLA2G5/BST1 |
| hsa00430 | Taurine and hypotaurine metabolism | 1/23 | 16/8577 | 4.21E-02 | FMO3 |
